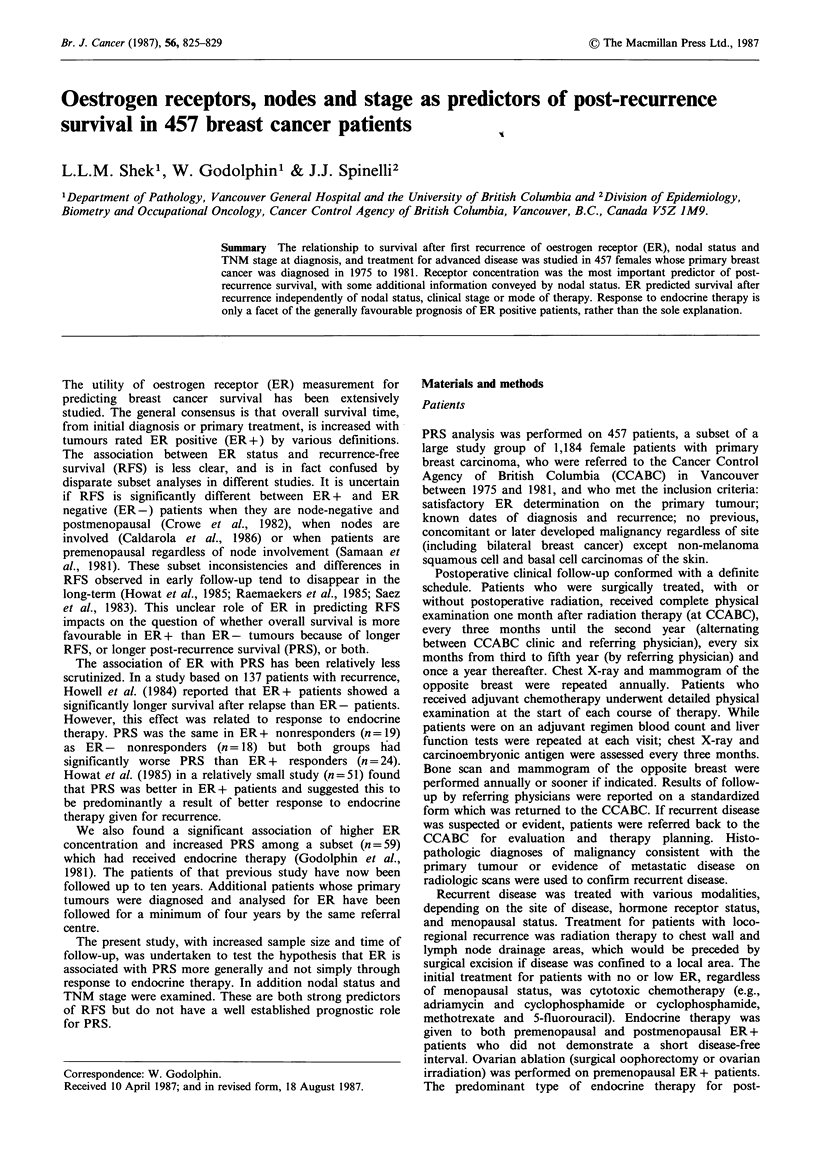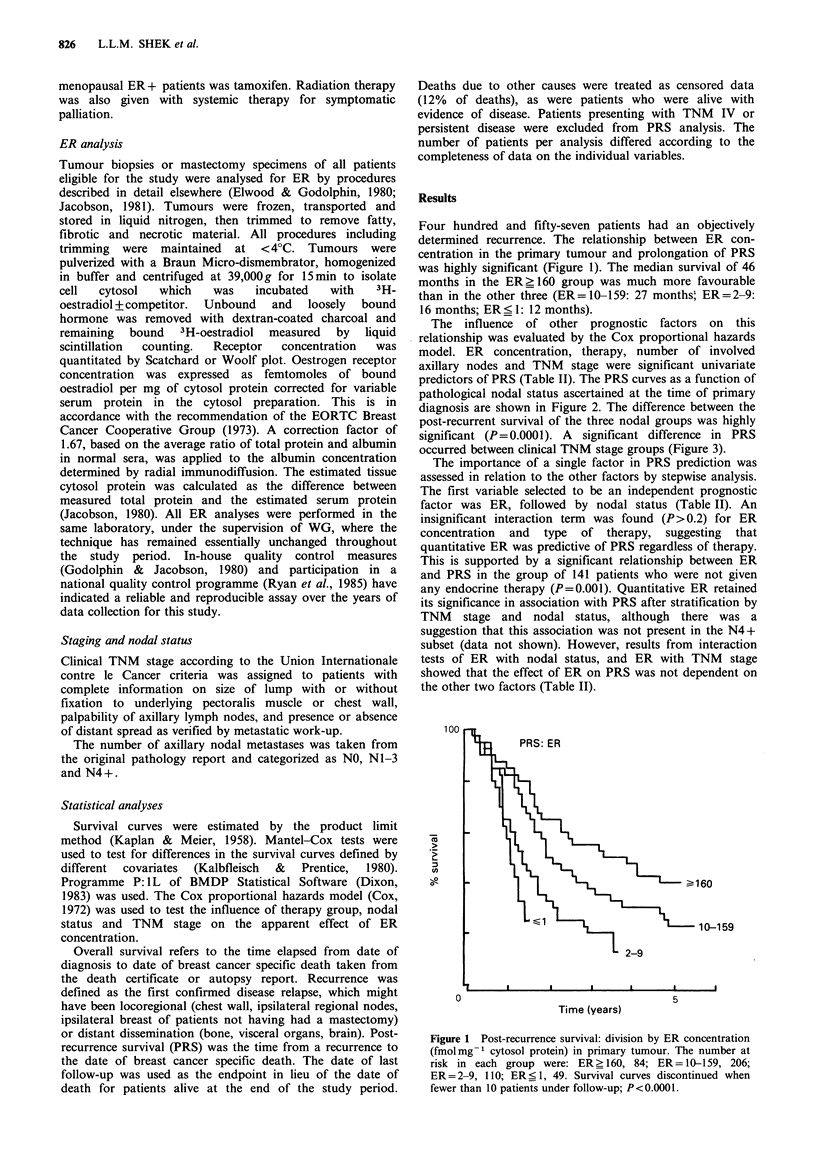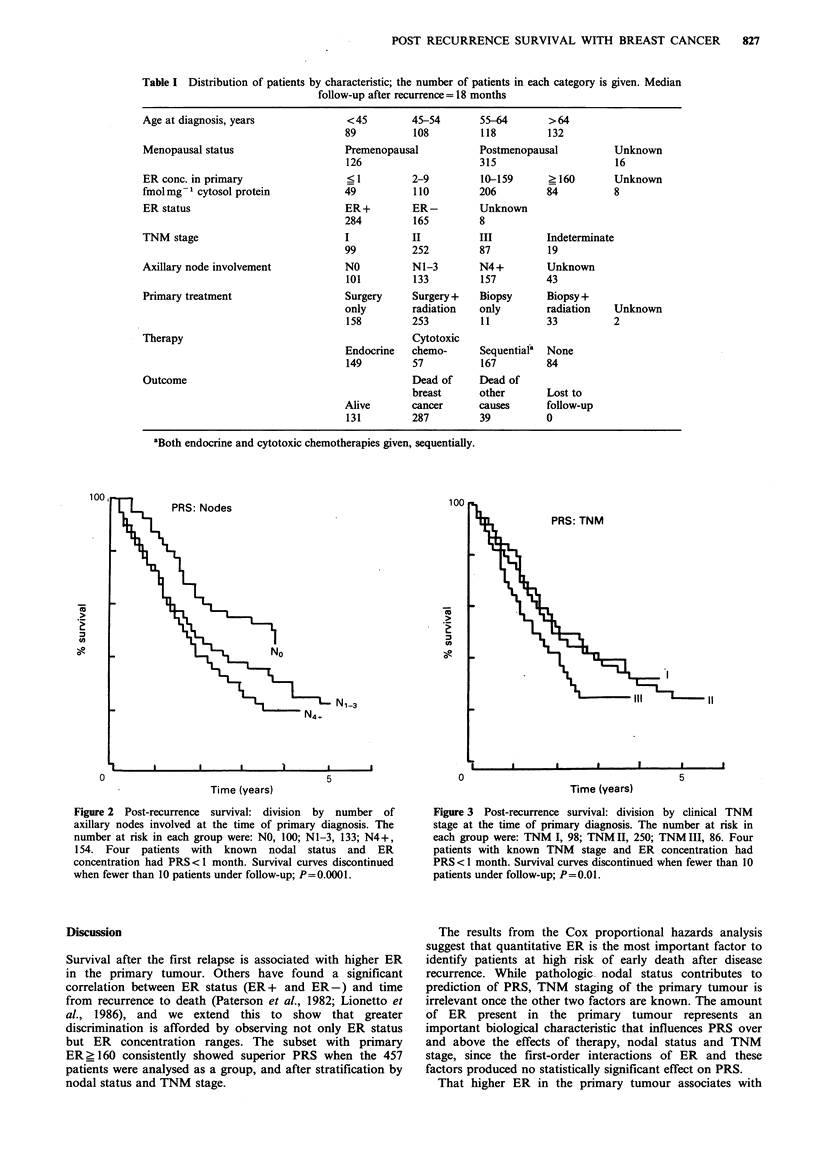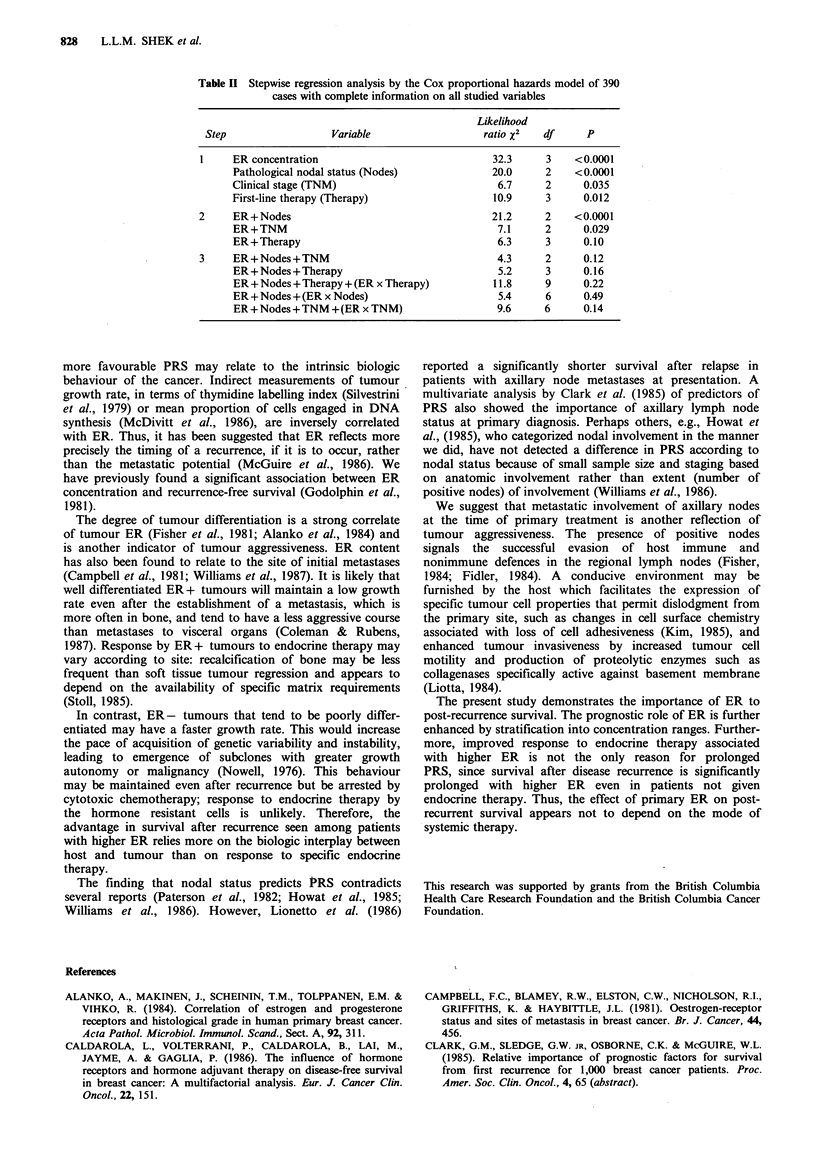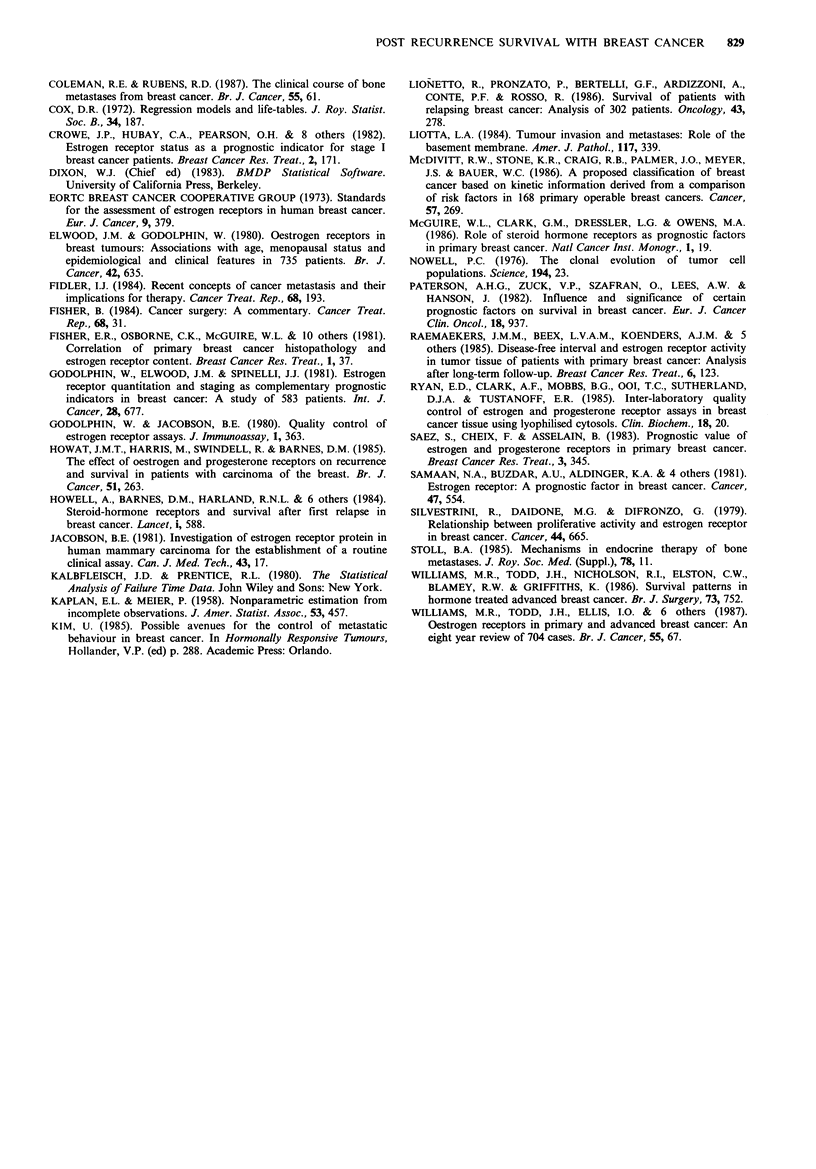# Oestrogen receptors, nodes and stage as predictors of post-recurrence survival in 457 breast cancer patients.

**DOI:** 10.1038/bjc.1987.298

**Published:** 1987-12

**Authors:** L. L. Shek, W. Godolphin, J. J. Spinelli

**Affiliations:** Department of Pathology, Vancouver General Hospital, B.C., Canada.

## Abstract

The relationship to survival after first recurrence of oestrogen receptor (ER), nodal status and TNM stage at diagnosis, and treatment for advanced disease was studied in 457 females whose primary breast cancer was diagnosed in 1975 to 1981. Receptor concentration was the most important predictor of post-recurrence survival, with some additional information conveyed by nodal status. ER predicted survival after recurrence independently of nodal status, clinical stage or mode of therapy. Response to endocrine therapy is only a facet of the generally favourable prognosis of ER positive patients, rather than the sole explanation.


Br. J. Cancer (1987), 56, 825–829 © The Macmillan Press Ltd., 1987

### Oestrogen receptors, nodes and stage as predictors of post-recurrence survival in 457 breast cancer patients

L.L.M. Shek[1], W. Godolphin[1] & J.J. Spinelli[2]

[1]*Department of Pathology, Vancouver General Hospital and the University of British Columbia and* [2]*Division of Epidemiology, Biometry and Occupational Oncology, Cancer Control Agency of British Columbia, Vancouver, B.C., Canada V5Z 1M9.*

**Summary** The relationship to survival after first recurrence of oestrogen receptor (ER), nodal status and TNM stage at diagnosis, and treatment for advanced disease was studied in 457 females whose primary breast cancer was diagnosed in 1975 to 1981. Receptor concentration was the most important predictor of post-recurrence survival, with some additional information conveyed by nodal status. ER predicted survival after recurrence independently of nodal status, clinical stage or mode of therapy. Response to endocrine therapy is only a facet of the generally favourable prognosis of ER positive patients, rather than the sole explanation.

The utility of oestrogen receptor (ER) measurement for predicting breast cancer survival has been extensively studied. The general consensus is that overall survival time, from initial diagnosis or primary treatment, is increased with tumours rated ER positive (ER+) by various definitions. The association between ER status and recurrence-free survival (RFS) is less clear, and is in fact confused by disparate subset analyses in different studies. It is uncertain if RFS is significantly different between ER+ and ER negative (ER−) patients when they are node-negative and postmenopausal (Crowe *et al.*, 1982), when nodes are involved (Caldarola *et al.*, 1986) or when patients are premenopausal regardless of node involvement (Samaan *et al.*, 1981). These subset inconsistencies and differences in RFS observed in early follow-up tend to disappear in the long-term (Howat *et al.*, 1985; Raemaekers *et al.*, 1985; Saez *et al.*, 1983). This unclear role of ER in predicting RFS impacts on the question of whether overall survival is more favourable in ER+ than ER− tumours because of longer RFS, or longer post-recurrence survival (PRS), or both.

The association of ER with PRS has been relatively less scrutinized. In a study based on 137 patients with recurrence, Howell *et al.* (1984) reported that ER+ patients showed a significantly longer survival after relapse than ER− patients. However, this effect was related to response to endocrine therapy. PRS was the same in ER+ nonresponders ($n=19$) as ER− nonresponders ($n=18$) but both groups had significantly worse PRS than ER+ responders ($n=24$). Howat *et al.* (1985) in a relatively small study ($n=51$) found that PRS was better in ER+ patients and suggested this to be predominantly a result of better response to endocrine therapy given for recurrence.

We also found a significant association of higher ER concentration and increased PRS among a subset ($n=59$) which had received endocrine therapy (Godolphin *et al.*, 1981). The patients of that previous study have now been followed up to ten years. Additional patients whose primary tumours were diagnosed and analysed for ER have been followed for a minimum of four years by the same referral centre.

The present study, with increased sample size and time of follow-up, was undertaken to test the hypothesis that ER is associated with PRS more generally and not simply through response to endocrine therapy. In addition nodal status and TNM stage were examined. These are both strong predictors of RFS but do not have a well established prognostic role for PRS.

Correspondence: W. Godolphin.
Received 10 April 1987; and in revised form, 18 August 1987.

### Materials and methods

### *Patients*

PRS analysis was performed on 457 patients, a subset of a large study group of 1,184 female patients with primary breast carcinoma, who were referred to the Cancer Control Agency of British Columbia (CCABC) in Vancouver between 1975 and 1981, and who met the inclusion criteria: satisfactory ER determination on the primary tumour; known dates of diagnosis and recurrence; no previous, concomitant or later developed malignancy regardless of site (including bilateral breast cancer) except non-melanoma squamous cell and basal cell carcinomas of the skin.

Postoperative clinical follow-up conformed with a definite schedule. Patients who were surgically treated, with or without postoperative radiation, received complete physical examination one month after radiation therapy (at CCABC), every three months until the second year (alternating between CCABC clinic and referring physician), every six months from third to fifth year (by referring physician) and once a year thereafter. Chest X-ray and mammogram of the opposite breast were repeated annually. Patients who received adjuvant chemotherapy underwent detailed physical examination at the start of each course of therapy. While patients were on an adjuvant regimen blood count and liver function tests were repeated at each visit; chest X-ray and carcinoembryonic antigen were assessed every three months. Bone scan and mammogram of the opposite breast were performed annually or sooner if indicated. Results of follow-up by referring physicians were reported on a standardized form which was returned to the CCABC. If recurrent disease was suspected or evident, patients were referred back to the CCABC for evaluation and therapy planning. Histopathologic diagnoses of malignancy consistent with the primary tumour or evidence of metastatic disease on radiologic scans were used to confirm recurrent disease.

Recurrent disease was treated with various modalities, depending on the site of disease, hormone receptor status, and menopausal status. Treatment for patients with locoregional recurrence was radiation therapy to chest wall and lymph node drainage areas, which would be preceded by surgical excision if disease was confined to a local area. The initial treatment for patients with no or low ER, regardless of menopausal status, was cytotoxic chemotherapy (e.g., adriamycin and cyclophosphamide or cyclophosphamide, methotrexate and 5-fluorouracil). Endocrine therapy was given to both premenopausal and postmenopausal ER+ patients who did not demonstrate a short disease-free interval. Ovarian ablation (surgical oophorectomy or ovarian irradiation) was performed on premenopausal ER+ patients. The predominant type of endocrine therapy for postmenopausal ER+ patients was tamoxifen. Radiation therapy was also given with systemic therapy for symptomatic palliation.

### *ER analysis*

Tumour biopsies or mastectomy specimens of all patients eligible for the study were analysed for ER by procedures described in detail elsewhere (Elwood & Godolphin, 1980; Jacobson, 1981). Tumours were frozen, transported and stored in liquid nitrogen, then trimmed to remove fatty, fibrotic and necrotic material. All procedures including trimming were maintained at <4°C. Tumours were pulverized with a Braun Micro-dismembrator, homogenized in buffer and centrifuged at 39,000$g$ for 15 min to isolate cell cytosol which was incubated with $^{3}$H-oestradiol ± competitor. Unbound and loosely bound hormone was removed with dextran-coated charcoal and remaining bound $^{3}$H-oestradiol measured by liquid scintillation counting. Receptor concentration was quantitated by Scatchard or Woolf plot. Oestrogen receptor concentration was expressed as femtomoles of bound oestradiol per mg of cytosol protein corrected for variable serum protein in the cytosol preparation. This is in accordance with the recommendation of the EORTC Breast Cancer Cooperative Group (1973). A correction factor of 1.67, based on the average ratio of total protein and albumin in normal sera, was applied to the albumin concentration determined by radial immunodiffusion. The estimated tissue cytosol protein was calculated as the difference between measured total protein and the estimated serum protein (Jacobson, 1980). All ER analyses were performed in the same laboratory, under the supervision of WG, where the technique has remained essentially unchanged throughout the study period. In-house quality control measures (Godolphin & Jacobson, 1980) and participation in a national quality control programme (Ryan *et al.*, 1985) have indicated a reliable and reproducible assay over the years of data collection for this study.

### *Staging and nodal status*

Clinical TNM stage according to the Union Internationale contre le Cancer criteria was assigned to patients with complete information on size of lump with or without fixation to underlying pectoralis muscle or chest wall, palpability of axillary lymph nodes, and presence or absence of distant spread as verified by metastatic work-up.

The number of axillary nodal metastases was taken from the original pathology report and categorized as N0, N1–3 and N4+.

### *Statistical analyses*

Survival curves were estimated by the product limit method (Kaplan & Meier, 1958). Mantel–Cox tests were used to test for differences in the survival curves defined by different covariates (Kalbfleisch & Prentice, 1980). Programme P:1L of BMDP Statistical Software (Dixon, 1983) was used. The Cox proportional hazards model (Cox, 1972) was used to test the influence of therapy group, nodal status and TNM stage on the apparent effect of ER concentration.

Overall survival refers to the time elapsed from date of diagnosis to date of breast cancer specific death taken from the death certificate or autopsy report. Recurrence was defined as the first confirmed disease relapse, which might have been locoregional (chest wall, ipsilateral regional nodes, ipsilateral breast of patients not having had a mastectomy) or distant dissemination (bone, visceral organs, brain). Post-recurrence survival (PRS) was the time from a recurrence to the date of breast cancer specific death. The date of last follow-up was used as the endpoint in lieu of the date of death for patients alive at the end of the study period. Deaths due to other causes were treated as censored data (12% of deaths), as were patients who were alive with evidence of disease. Patients presenting with TNM IV or persistent disease were excluded from PRS analysis. The number of patients per analysis differed according to the completeness of data on the individual variables.

### Results

Four hundred and fifty-seven patients had an objectively determined recurrence. The relationship between ER concentration in the primary tumour and prolongation of PRS was highly significant (Figure 1). The median survival of 46 months in the ER ≧ 160 group was much more favourable than in the other three (ER = 10–159: 27 months; ER = 2–9: 16 months; ER ≦ 1: 12 months).

The influence of other prognostic factors on this relationship was evaluated by the Cox proportional hazards model. ER concentration, therapy, number of involved axillary nodes and TNM stage were significant univariate predictors of PRS (Table II). The PRS curves as a function of pathological nodal status ascertained at the time of primary diagnosis are shown in Figure 2. The difference between the post-recurrent survival of the three nodal groups was highly significant ($P$ = 0.0001). A significant difference in PRS occurred between clinical TNM stage groups (Figure 3).

The importance of a single factor in PRS prediction was assessed in relation to the other factors by stepwise analysis. The first variable selected to be an independent prognostic factor was ER, followed by nodal status (Table II). An insignificant interaction term was found ($P$ > 0.2) for ER concentration and type of therapy, suggesting that quantitative ER was predictive of PRS regardless of therapy. This is supported by a significant relationship between ER and PRS in the group of 141 patients who were not given any endocrine therapy ($P$ = 0.001). Quantitative ER retained its significance in association with PRS after stratification by TNM stage and nodal status, although there was a suggestion that this association was not present in the N4+ subset (data not shown). However, results from interaction tests of ER with nodal status, and ER with TNM stage showed that the effect of ER on PRS was not dependent on the other two factors (Table II).

**Figure 1** Post-recurrence survival: division by ER concentration (fmol mg$^{-1}$ cytosol protein) in primary tumour. The number at risk in each group were: ER ≧ 160, 84; ER = 10–159, 206; ER = 2–9, 110; ER ≦ 1, 49. Survival curves discontinued when fewer than 10 patients under follow-up; $P$ < 0.0001.

**Table I** Distribution of patients by characteristic; the number of patients in each category is given. Median follow-up after recurrence = 18 months

| | | | | | |
|---|---|---|---|---|---|
| Age at diagnosis, years | <45<br>89 | 45–54<br>108 | 55–64<br>118 | >64<br>132 | |
| Menopausal status | Premenopausal<br>126 | | Postmenopausal<br>315 | | Unknown<br>16 |
| ER conc. in primary fmol mg$^{-1}$ cytosol protein | ≦1<br>49 | 2–9<br>110 | 10–159<br>206 | ≧160<br>84 | Unknown<br>8 |
| ER status | ER+<br>284 | ER−<br>165 | Unknown<br>8 | | |
| TNM stage | I<br>99 | II<br>252 | III<br>87 | Indeterminate<br>19 | |
| Axillary node involvement | N0<br>101 | N1-3<br>133 | N4+<br>157 | Unknown<br>43 | |
| Primary treatment | Surgery only<br>158 | Surgery+ radiation<br>253 | Biopsy only<br>11 | Biopsy+ radiation<br>33 | Unknown<br>2 |
| Therapy | Endocrine<br>149 | Cytotoxic chemo-<br>57 | Sequential[a]<br>167 | None<br>84 | |
| Outcome | Alive<br>131 | Dead of breast cancer<br>287 | Dead of other causes<br>39 | Lost to follow-up<br>0 | |

[a]Both endocrine and cytotoxic chemotherapies given, sequentially.

**Figure 2** Post-recurrence survival: division by number of axillary nodes involved at the time of primary diagnosis. The number at risk in each group were: N0, 100; N1-3, 133; N4+, 154. Four patients with known nodal status and ER concentration had PRS<1 month. Survival curves discontinued when fewer than 10 patients under follow-up; $P$=0.0001.

**Figure 3** Post-recurrence survival: division by clinical TNM stage at the time of primary diagnosis. The number at risk in each group were: TNM I, 98; TNM II, 250; TNM III, 86. Four patients with known TNM stage and ER concentration had PRS<1 month. Survival curves discontinued when fewer than 10 patients under follow-up; $P$=0.01.

### Discussion

Survival after the first relapse is associated with higher ER in the primary tumour. Others have found a significant correlation between ER status (ER+ and ER−) and time from recurrence to death (Paterson *et al.*, 1982; Lionetto *et al.*, 1986), and we extend this to show that greater discrimination is afforded by observing not only ER status but ER concentration ranges. The subset with primary ER≧160 consistently showed superior PRS when the 457 patients were analysed as a group, and after stratification by nodal status and TNM stage.

The results from the Cox proportional hazards analysis suggest that quantitative ER is the most important factor to identify patients at high risk of early death after disease recurrence. While pathologic nodal status contributes to prediction of PRS, TNM staging of the primary tumour is irrelevant once the other two factors are known. The amount of ER present in the primary tumour represents an important biological characteristic that influences PRS over and above the effects of therapy, nodal status and TNM stage, since the first-order interactions of ER and these factors produced no statistically significant effect on PRS.

That higher ER in the primary tumour associates with

**Table II** Stepwise regression analysis by the Cox proportional hazards model of 390 cases with complete information on all studied variables

| *Step* | *Variable* | *Likelihood ratio* $\chi^2$ | *df* | *P* |
|---|---|---|---|---|
| 1 | ER concentration | 32.3 | 3 | <0.0001 |
| | Pathological nodal status (Nodes) | 20.0 | 2 | <0.0001 |
| | Clinical stage (TNM) | 6.7 | 2 | 0.035 |
| | First-line therapy (Therapy) | 10.9 | 3 | 0.012 |
| 2 | ER+Nodes | 21.2 | 2 | <0.0001 |
| | ER+TNM | 7.1 | 2 | 0.029 |
| | ER+Therapy | 6.3 | 3 | 0.10 |
| 3 | ER+Nodes+TNM | 4.3 | 2 | 0.12 |
| | ER+Nodes+Therapy | 5.2 | 3 | 0.16 |
| | ER+Nodes+Therapy+(ER x Therapy) | 11.8 | 9 | 0.22 |
| | ER+Nodes+(ER x Nodes) | 5.4 | 6 | 0.49 |
| | ER+Nodes+TNM+(ER x TNM) | 9.6 | 6 | 0.14 |

more favourable PRS may relate to the intrinsic biologic behaviour of the cancer. Indirect measurements of tumour growth rate, in terms of thymidine labelling index (Silvestrini *et al.*, 1979) or mean proportion of cells engaged in DNA synthesis (McDivitt *et al.*, 1986), are inversely correlated with ER. Thus, it has been suggested that ER reflects more precisely the timing of a recurrence, if it is to occur, rather than the metastatic potential (McGuire *et al.*, 1986). We have previously found a significant association between ER concentration and recurrence-free survival (Godolphin *et al.*, 1981).

The degree of tumour differentiation is a strong correlate of tumour ER (Fisher *et al.*, 1981; Alanko *et al.*, 1984) and is another indicator of tumour aggressiveness. ER content has also been found to relate to the site of initial metastases (Campbell *et al.*, 1981; Williams *et al.*, 1987). It is likely that well differentiated ER+ tumours will maintain a low growth rate even after the establishment of a metastasis, which is more often in bone, and tend to have a less aggressive course than metastases to visceral organs (Coleman & Rubens, 1987). Response by ER+ tumours to endocrine therapy may vary according to site: recalcification of bone may be less frequent than soft tissue tumour regression and appears to depend on the availability of specific matrix requirements (Stoll, 1985).

In contrast, ER− tumours that tend to be poorly differentiated may have a faster growth rate. This would increase the pace of acquisition of genetic variability and instability, leading to emergence of subclones with greater growth autonomy or malignancy (Nowell, 1976). This behaviour may be maintained even after recurrence but be arrested by cytotoxic chemotherapy; response to endocrine therapy by the hormone resistant cells is unlikely. Therefore, the advantage in survival after recurrence seen among patients with higher ER relies more on the biologic interplay between host and tumour than on response to specific endocrine therapy.

The finding that nodal status predicts PRS contradicts several reports (Paterson *et al.*, 1982; Howat *et al.*, 1985; Williams *et al.*, 1986). However, Lionetto *et al.* (1986) reported a significantly shorter survival after relapse in patients with axillary node metastases at presentation. A multivariate analysis by Clark *et al.* (1985) of predictors of PRS also showed the importance of axillary lymph node status at primary diagnosis. Perhaps others, e.g., Howat *et al.*, (1985), who categorized nodal involvement in the manner we did, have not detected a difference in PRS according to nodal status because of small sample size and staging based on anatomic involvement rather than extent (number of positive nodes) of involvement (Williams *et al.*, 1986).

We suggest that metastatic involvement of axillary nodes at the time of primary treatment is another reflection of tumour aggressiveness. The presence of positive nodes signals the successful evasion of host immune and nonimmune defences in the regional lymph nodes (Fisher, 1984; Fidler, 1984). A conducive environment may be furnished by the host which facilitates the expression of specific tumour cell properties that permit dislodgment from the primary site, such as changes in cell surface chemistry associated with loss of cell adhesiveness (Kim, 1985), and enhanced tumour invasiveness by increased tumour cell motility and production of proteolytic enzymes such as collagenases specifically active against basement membrane (Liotta, 1984).

The present study demonstrates the importance of ER to post-recurrence survival. The prognostic role of ER is further enhanced by stratification into concentration ranges. Furthermore, improved response to endocrine therapy associated with higher ER is not the only reason for prolonged PRS, since survival after disease recurrence is significantly prolonged with higher ER even in patients not given endocrine therapy. Thus, the effect of primary ER on post-recurrent survival appears not to depend on the mode of systemic therapy.

This research was supported by grants from the British Columbia Health Care Research Foundation and the British Columbia Cancer Foundation.

### References

ALANKO, A., MAKINEN, J., SCHEININ, T.M., TOLPPANEN, E.M. & VIHKO, R. (1984). Correlation of estrogen and progesterone receptors and histological grade in human primary breast cancer. *Acta Pathol. Microbiol. Immunol. Scand.*, Sect. A, **92**, 311.

CALDAROLA, L., VOLTERRANI, P., CALDAROLA, B., LAI, M., JAYME, A. & GAGLIA, P. (1986). The influence of hormone receptors and hormone adjuvant therapy on disease-free survival in breast cancer: A multifactorial analysis. *Eur. J. Cancer Clin. Oncol.*, **22**, 151.

CAMPBELL, F.C., BLAMEY, R.W., ELSTON, C.W., NICHOLSON, R.I., GRIFFITHS, K. & HAYBITTLE, J.L. (1981). Oestrogen-receptor status and sites of metastasis in breast cancer. *Br. J. Cancer*, **44**, 456.

CLARK, G.M., SLEDGE, G.W. JR, OSBORNE, C.K. & MCGUIRE, W.L. (1985). Relative importance of prognostic factors for survival from first recurrence for 1,000 breast cancer patients. *Proc. Amer. Soc. Clin. Oncol.*, **4**, 65 (*abstract*).

COLEMAN, R.E. & RUBENS, R.D. (1987). The clinical course of bone metastases from breast cancer. *Br. J. Cancer*, **55**, 61.

COX, D.R. (1972). Regression models and life-tables. *J. Roy. Statist. Soc. B.*, **34**, 187.

CROWE, J.P., HUBAY, C.A., PEARSON, O.H. & 8 others (1982). Estrogen receptor status as a prognostic indicator for stage I breast cancer patients. *Breast Cancer Res. Treat.*, **2**, 171.

DIXON, W.J. (Chief ed) (1983). *BMDP Statistical Software*. University of California Press, Berkeley.

EORTC BREAST CANCER COOPERATIVE GROUP (1973). Standards for the assessment of estrogen receptors in human breast cancer. *Eur. J. Cancer*, **9**, 379.

ELWOOD, J.M. & GODOLPHIN, W. (1980). Oestrogen receptors in breast tumours: Associations with age, menopausal status and epidemiological and clinical features in 735 patients. *Br. J. Cancer*, **42**, 635.

FIDLER, I.J. (1984). Recent concepts of cancer metastasis and their implications for therapy. *Cancer Treat. Rep.*, **68**, 193.

FISHER, B. (1984). Cancer surgery: A commentary. *Cancer Treat. Rep.*, **68**, 31.

FISHER, E.R., OSBORNE, C.K., McGUIRE, W.L. & 10 others (1981). Correlation of primary breast cancer histopathology and estrogen receptor content. *Breast Cancer Res. Treat.*, **1**, 37.

GODOLPHIN, W., ELWOOD, J.M. & SPINELLI, J.J. (1981). Estrogen receptor quantitation and staging as complementary prognostic indicators in breast cancer: A study of 583 patients. *Int. J. Cancer*, **28**, 677.

GODOLPHIN, W. & JACOBSON, B.E. (1980). Quality control of estrogen receptor assays. *J. Immunoassay*, **1**, 363.

HOWAT, J.M.T., HARRIS, M., SWINDELL, R. & BARNES, D.M. (1985). The effect of oestrogen and progesterone receptors on recurrence and survival in patients with carcinoma of the breast. *Br. J. Cancer*, **51**, 263.

HOWELL, A., BARNES, D.M., HARLAND, R.N.L. & 6 others (1984). Steroid-hormone receptors and survival after first relapse in breast cancer. *Lancet*, **i**, 588.

JACOBSON, B.E. (1981). Investigation of estrogen receptor protein in human mammary carcinoma for the establishment of a routine clinical assay. *Can. J. Med. Tech.*, **43**, 17.

KALBFLEISCH, J.D. & PRENTICE, R.L. (1980). *The Statistical Analysis of Failure Time Data*. John Wiley and Sons: New York.

KAPLAN, E.L. & MEIER, P. (1958). Nonparametric estimation from incomplete observations. *J. Amer. Statist. Assoc.*, **53**, 457.

KIM, U. (1985). Possible avenues for the control of metastatic behaviour in breast cancer. In *Hormonally Responsive Tumours*, Hollander, V.P. (ed) p. 288. Academic Press: Orlando.

LIOÑETTO, R., PRONZATO, P., BERTELLI, G.F., ARDIZZONI, A., CONTE, P.F. & ROSSO, R. (1986). Survival of patients with relapsing breast cancer: Analysis of 302 patients. *Oncology*, **43**, 278.

LIOTTA, L.A. (1984). Tumour invasion and metastases: Role of the basement membrane. *Amer. J. Pathol.*, **117**, 339.

McDIVITT, R.W., STONE, K.R., CRAIG, R.B., PALMER, J.O., MEYER, J.S. & BAUER, W.C. (1986). A proposed classification of breast cancer based on kinetic information derived from a comparison of risk factors in 168 primary operable breast cancers. *Cancer*, **57**, 269.

McGUIRE, W.L., CLARK, G.M., DRESSLER, L.G. & OWENS, M.A. (1986). Role of steroid hormone receptors as prognostic factors in primary breast cancer. *Natl Cancer Inst. Monogr.*, **1**, 19.

NOWELL, P.C. (1976). The clonal evolution of tumor cell populations. *Science*, **194**, 23.

PATERSON, A.H.G., ZUCK, V.P., SZAFRAN, O., LEES, A.W. & HANSON, J. (1982). Influence and significance of certain prognostic factors on survival in breast cancer. *Eur. J. Cancer Clin. Oncol.*, **18**, 937.

RAEMAEKERS, J.M.M., BEEX, L.V.A.M., KOENDERS, A.J.M. & 5 others (1985). Disease-free interval and estrogen receptor activity in tumor tissue of patients with primary breast cancer: Analysis after long-term follow-up. *Breast Cancer Res. Treat.*, **6**, 123.

RYAN, E.D., CLARK, A.F., MOBBS, B.G., OOI, T.C., SUTHERLAND, D.J.A. & TUSTANOFF, E.R. (1985). Inter-laboratory quality control of estrogen and progesterone receptor assays in breast cancer tissue using lyophilised cytosols. *Clin. Biochem.*, **18**, 20.

SAEZ, S., CHEIX, F. & ASSELAIN, B. (1983). Prognostic value of estrogen and progesterone receptors in primary breast cancer. *Breast Cancer Res. Treat.*, **3**, 345.

SAMAAN, N.A., BUZDAR, A.U., ALDINGER, K.A. & 4 others (1981). Estrogen receptor: A prognostic factor in breast cancer. *Cancer*, **47**, 554.

SILVESTRINI, R., DAIDONE, M.G. & DIFRONZO, G. (1979). Relationship between proliferative activity and estrogen receptor in breast cancer. *Cancer*, **44**, 665.

STOLL, B.A. (1985). Mechanisms in endocrine therapy of bone metastases. *J. Roy. Soc. Med.* (Suppl.), **78**, 11.

WILLIAMS, M.R., TODD, J.H., NICHOLSON, R.I., ELSTON, C.W., BLAMEY, R.W. & GRIFFITHS, K. (1986). Survival patterns in hormone treated advanced breast cancer. *Br. J. Surgery*, **73**, 752.

WILLIAMS, M.R., TODD, J.H., ELLIS, I.O. & 6 others (1987). Oestrogen receptors in primary and advanced breast cancer: An eight year review of 704 cases. *Br. J. Cancer*, **55**, 67.